# 13.2% efficiency Si nanowire/PEDOT:PSS hybrid solar cell using a transfer-imprinted Au mesh electrode

**DOI:** 10.1038/srep12093

**Published:** 2015-07-15

**Authors:** Kwang-Tae Park, Han-Jung Kim, Min-Joon Park, Jun-Ho Jeong, Jihye Lee, Dae-Geun Choi, Jung-Ho Lee, Jun-Hyuk Choi

**Affiliations:** 1 Department of Nano Manufacturing Technology, Korea Institute of Machinery and Materials (KIMM), Daejeon, 305-343, Korea; 2 Department of Chemical Engineering, Hanyang University, Ansan, 426-791, Korea; 3 Department of Nano-Mechatronics, University of Science and Technology, Daejeon, 305-350, Korea

## Abstract

In recent years, inorganic/organic hybrid solar cell concept has received growing attention for alternative energy solution because of the potential for facile and low-cost fabrication and high efficiency. Here, we report highly efficient hybrid solar cells based on silicon nanowires (SiNWs) and poly(3,4-ethylenedioxythiophene):poly(styrenesulfonate) (PEDOT:PSS) using transfer-imprinted metal mesh front electrodes. Such a structure increases the optical absorption and shortens the carrier transport distance, thus, it greatly increases the charge carrier collection efficiency. Compared with hybrid cells formed using indium tin oxide (ITO) electrodes, we find an increase in power conversion efficiency from 5.95% to 13.2%, which is attributed to improvements in both the electrical and optical properties of the Au mesh electrode. Our fabrication strategy for metal mesh electrode is suitable for the large-scale fabrication of flexible transparent electrodes, paving the way towards low-cost, high-efficiency, flexible solar cells.

In recent years, photovoltaics have attracted growing research interest for applications in renewable energy technology due to rising concerns about climate change and the sustainability of fossil fuels. Silicon (Si) nanostructures have been investigated as potential candidates for next-generation solar cells because of their efficient light harvesting and carrier collection capability despite reduced Si usage^1^,^2^,^3^,^4^,^5^,^6^. However, all-inorganic solar cells based on Si nanostructures require energy-intensive semiconductor processes, including high-temperature thermal diffusion, thermal annealing of the electrodes, and high-vacuum chemical deposition processes. Furthermore, the reported experimental efficiencies remain significantly lower than the estimated theoretical efficiency^7^ as well as the efficiency of conventional Si solar cells. To resolve these problems, inorganic/organic hybrid solar cells have been proposed, which are composed of an *n*-type crystalline Si nanostructure base and an organic poly(3,4-ethylenedioxythiophene):poly(styrenesulfonate) (PEDOT:PSS) hole-transport/emitter layer^8^,^9^,^10^,^11^,^12^,^13^,^14^,^15^,^16^,^17^,^18^,^19^. This architecture has the potential to combine the broadband optical absorption capability of the Si nanostructures with solution-based processes for simple and low-cost production.

Some recent reports have demonstrated technological progress toward high-efficiency Si/PEDOT:PSS hybrid solar cells. The general strategy for hybrid solar cells is to use unique nanostructures to absorb the broad solar spectrum, including nanowires (NWs)^8^,^9^, nanocones^10^, nanoholes^11^, nanotubes^12^, and nanopyramids^13^. He *et al.* designed a SiNW/pyramid binary structure with a large heterojunction area and excellent optical absorption using relatively short SiNWs^14^. A highly doped back surface^10^ or wide-band-gap organic semiconductor, such as 8-hydroxyquinolinolato-lithium (Liq)^15^, can be introduced between the Si substrate and the back electrode to form a back surface field. Yu *et al.* reported a power conversion efficiency (PCE) of 13% for a SiNW/PEDOT:PSS hybrid solar cell using an intermediate 1,1-bis[(di-4-tolylamino)phenyl]cyclohexane (TAPC) layer to mitigate interface recombination^16^.

The PCE of state-of-the-art hybrid solar cells is in the range of 10–13%. Strategies that have been followed to achieve high efficiency include modifying the nanostructures^8^,^9^,^10^,^11^,^12^,^13^,^14^, modifying the interface between Si and PEDOT:PSS^16^,^17^,^18^, and engineering the back contact^10^,^15^. However, to the best of our knowledge, there have been no reports on realizing the importance of the front electrode for optimized optical and electrical properties, which is critical for a high PCE. Indium tin oxide (ITO) is the most widely used transparent electrode because of its high optical transmittance and low sheet resistance; however, it has a number of shortcomings for applications in photovoltaics, including the rising cost of indium and performance degradation due to stability issues of the PEDOT:PSS/ITO interface^19^,^20^,^21^. Metal grids do not suffer from these stability issues; however, they typically shade 8–10% of the front surface area because of the stencil-based deposition of material through shadow masks that is typically used in the fabrication process^6^. Therefore, there is a strong need for the alternative approach for the front electrode.

Here, we use a highly conductive, transparent, and stable gold (Au) mesh electrode, formed using a metal-transfer imprinting method, to fabricate SiNW/PEDOT:PSS hybrid solar cells. Compared with typical metal grids via stencil method, the reduced pitch and width of the Au mesh electrode result in higher short-circuit current density *J*_*sc*_ and lower collection losses of the photogenerated carriers. As a result, we demonstrate hybrid solar cells with a record PCE of 13.2%, a short-circuit current density of *J*_*sc*_ = 36.03 mA/cm^2^, an open-circuit voltage of *V*_*oc*_ = 539.2 mV, and a fill factor (*FF*) of 67.8%. In particular, remarkably high quantum efficiency was observed at blue wavelengths, indicating efficient collection of photogenerated charge carriers in the SiNWs.

## Results

**Figure 1:**
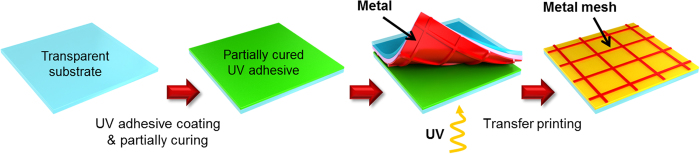
Metal transfer imprinting. A schematic illustration of the fabrication process for the mesh electrodes on transparent substrates.

Figure 1 shows a schematic illustration of the fabrication process for the uniform metal mesh electrode on transparent substrates (glass or polyethylene terephthalate (PET) film) using an ultraviolet (UV)-curable polyurethane acrylate (PUA) stamp. Au mesh electrodes were fabricated using molding, metal evaporation, and partial curing, as shown in Fig. 1. We demonstrated that the metal mesh structures have long-term stability and can be fabricated via a facile transfer imprinting method employing partial curing kinetics (see the Methods and Supplementary for further details).

**Figure 2:**
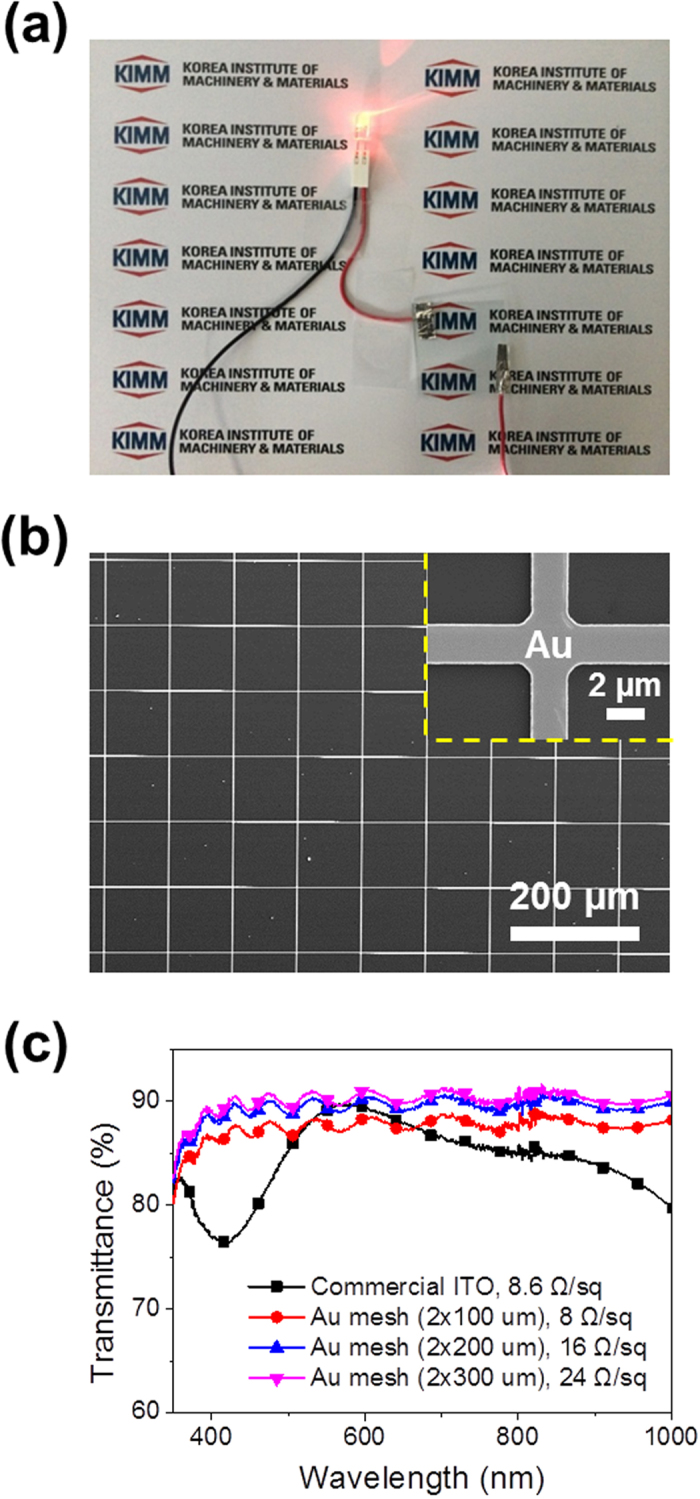
Transfer-imprinted Au mesh electrodes. (**a**) A photograph of a red light-emitting diode in operation using an Au mesh electrode. (**b**) An FESEM image of an Au mesh pattern, with a line-width of 2 μm and pitch of 100 μm. The inset in panel (**b**) shows a high-magnification SEM image. (**c**) Transmittance spectra of various Au mesh and ITO electrodes.

Figure 2a shows an image of the transfer-imprinted Au mesh electrode. The photograph shows high transparency as the symbol mark (KIMM) in the background can be clearly seen through the Au mesh electrode. The morphology of the Au mesh was characterized using field-emission scanning electron microscopy (FE-SEM, Hitachi S-4800). Figure 2b and S1 show the three different Au mesh structures, which were 150 nm thick with a 2-μm line width, and where the line pitch was varied in the range 100–300 μm. The inset in Fig. 2b shows a high-magnification SEM image of the same sample. The transmittance and sheet resistance of the Au mesh electrodes can be easily tuned by varying the thickness of the metal layers, as well as the line width and line pitch. The samples used in this study exhibited excellent uniformity of the mesh over a large area (6 × 6 cm^2^). This indicates that the metal-transfer imprinting method can be used for large-scale fabrication of transparent electrodes.

To evaluate the performance of the Au meshes as front electrodes for solar cells, transmittance measurements were carried out at wavelengths in the range 350–1000 nm using a UV–visible (UV–vis)/near-infrared (NIR) spectrophotometer (Carry 5000, Varian) equipped with an integrating sphere to account for the total (i.e., diffuse and specular) transmitted light from the sample. A four-point probe method was used to characterize the sheet resistance to avoid the effects of contact resistance between the Au mesh electrode and the probe tip. Figure 2c shows a comparison of the transmittance spectra of the Au mesh and a conventional, commercially available ITO electrode; ideally, large optical transmittance is required for high overall efficiency. Compared with the ITO electrode, which had a sheet resistance of 8.6 Ω/sq, the Au mesh electrodes exhibited large and broadband flat optical transmittance in the visible and NIR wavelength ranges, which is attributed to the large scattering and open spaces between the meshes^22^. The flat transmittance spectrum can boost the application of the Au mesh electrodes for solar cells through utilization of the broad solar spectrum. Also note that the transmittance decreased with decreasing line pitch, which is expected based on the increase in the total shadowing area per unit area. With a metal film structure, the total shadowing area is equal to the projected sample area. As the line pitch of Au meshes decreased, the sheet resistance also decreased. In other words, the electrical conductivity of the Au mesh electrode strongly depends on the line pitch size. A figure of merit (FOM) for the transparent electrodes can be calculated as follows^23^,^24^:


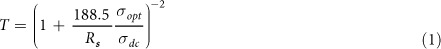


where *R*_*s*_ is the sheet resistance, *T* is the transmittance at 550 nm, and ***σ***_***dc***_**/*****σ***_***opt***_ is the ratio of the electrical conductance to the optical conductance. We find FOMs of 351, 210, and 147 for our Au mesh electrodes with line pitches of 100, 200, and 300 μm, respectively. The minimum FOM value that is typically required for transparent electrode applications is 35^23^,^25^. Therefore, our Au mesh electrodes are sufficiently adaptable to the hybrid solar cells.

**Figure 3:**
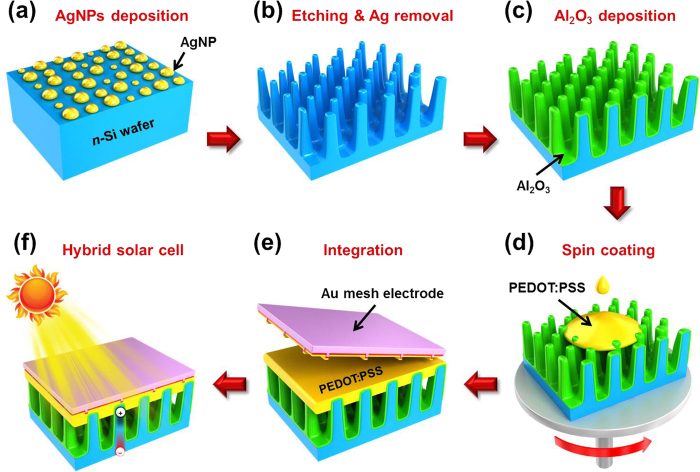
SiNW/PEDOT:PSS hybrid solar cell. An overview of the fabrication process for the SiNW/PEDOT:PSS hybrid solar cells. (**a**) AgNPs were precipitated using a galvanic displacement reaction. (**b**) SiNWs were allowed to form at room temperature for 20 s using a mixture of 4.8 M HF and 0.6 M H_2_O_2_. (c) An Al_2_O_3_ thin film was deposited using ALD to form a passivation layer. (**d**) PEDOT:PSS solution was spin coated onto both the SiNW array and the Au mesh electrode. (**e**) The PEDOT:PSS-coated Au mesh electrode and the SiNW array were directly pressed together and annealed. (**f**) A resulting SiNW/PEDOT:PSS hybrid solar cell.

Figure 3 shows an overview of the process used to fabricate the SiNW/PEDOT:PSS hybrid solar cells. We used one-step metal-assisted chemical etching (MACE) to form SiNWs, as described elsewhere^6^. Silver nanoparticles (AgNPs) were precipitated uniformly for 10 s from a galvanic displacement reaction employing an aqueous solution of HF (4.8 M) and AgNO_3_ (0.005 M), as shown in Fig. 3a. Because the redox potential of Ag^ + ^/Ag^0^ lies below the valence band of Si, electrons can be expected to transfer from the Si to the Ag^ + ^ions in the vicinity of the Si surface, leading to the deposition of numerous nano-sized Ag nuclei^26^. Following cleaning, SiNWs were allowed to form at room temperature for 20 s using a mixed solution of HF (4.8 M) and H_2_O_2_ (0.6 M). Residual AgNPs were then removed using concentrated nitric acid for 20 min, and the SiNWs were rinsed with de-ionized (DI) water, as shown in Fig. 3b. Increased carrier recombination at the surface or interface of the SiNWs due to their enlarged surface to volume ratio is a major obstacle in achieving highly efficient solar cells. To reduce carrier recombination at the nanotextured Si surface, a thin film of aluminum oxide (Al_2_O_3_) was deposited to form a passivation layer using atomic layer deposition (ALD), which can achieve conformal thin films on high-aspect-ratio surfaces such as an array of SiNWs, as shown in Fig. 3c^27^. The thickness of the Al_2_O_3_ passivation layer should not be too thick (>2.5 nm), because a thick passivation layer could create an insulating barrier for electrical transport, thereby deteriorating the solar cell performance^28^,^29^. A highly conductive PEDOT:PSS (<850 S/cm) solution mixed with 5 wt.% dimethylsulfoxide (DMSO) and 0.2 wt.% surfactant was spin-coated onto both the array of SiNWs and the Au mesh electrode at 6000 rpm for 60 s (Fig. 3d). The PEDOT:PSS-coated Au mesh electrode and SiNWs array were then directly pressed together and annealed at 150 °C for 10 min in a nitrogen atmosphere, as shown in Fig. 3e. Because PEDOT:PSS is a *p*-type organic semiconductor, *n*-SiNW/PEDOT:PSS forms a Schottky junction, which replace the expensive Si *p*-*n* junctions found in co*n*ventional Si solar cells. Figure 3f shows the SiNW/PEDOT:PSS hybrid solar cell with the transfer-imprinted Au mesh front electrode.

**Figure 4:**
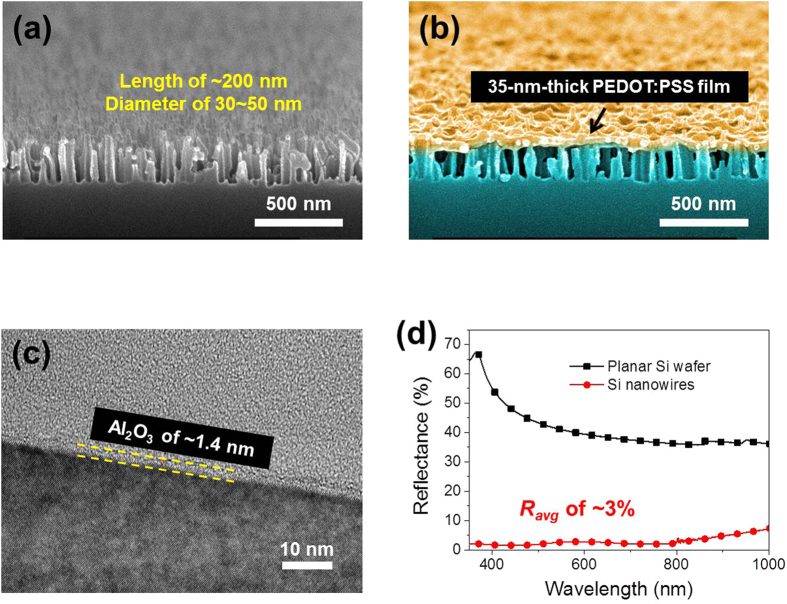
SiNW arrays. (**a**) The tilted cross-sectional SEM images of the 200-nm-long SiNWs. (**b**) The corresponding morphology after coating with the PEDOT:PSS solution. (**c**) A high-resolution TEM image of the Al_2_O_3_ layer on the crystalline Si sample. (**d**) Reflectance spectra of the 200 nm-long SiNWs before being coated with PEDOT:PSS, along with the Si wafer.

SiNWs array with average length of 200 nm and diameter of 30–50 nm was fabricated by chemical etching for 20 s. Figure 4a,b show the tilted cross-sectional SEM images of the SiNW array and their corresponding morphologies after coating with PEDOT:PSS solution, respectively. The density and diameter were determined by the deposition time of the AgNPs, and the length of the SiNW array could be controlled by varying the etching time. Because of the short diffusion length (≤10 nm) of the PEDOT:PSS conducting polymer^9^, a thin layer of the PEDOT:PSS is required for both efficient photocarrier generation and collection of charge carriers. As shown in Fig. 4b, the gaps between the SiNWs were in the range 30–60 nm; thus, the long-chain polymer PEDOT:PSS could not penetrate to the bottom of the array of SiNWs and therefore formed a continuous ~35-nm-thick film on top of the array of SiNWs. Field emission transmission electron microscopy (FE-TEM, FEI Tecnai F30 Super-Twin) at 300 kV was used to characterize the SiNWs passivated with the Al_2_O_3_ thin film; the sample was prepared using conventional mechanical polishing. Figure 4c shows a high-resolution TEM image of the Al_2_O_3_ layer on the crystalline Si sample. The Al_2_O_3_ layer was approximately 1.4 nm thick, which shows that a native oxide layer did not form during the six ALD cycles used to deposit the Al_2_O_3_. The Al_2_O_3_ film was uniformly coated onto the SiNWs, which can be expected to lead to lower surface recombination. Figure 4d shows the reflectance spectra of the 200-nm-long SiNWs before they were coated with PEDOT:PSS, measured using the integrating sphere. The reflectance of a planar Si wafer is also shown for comparison. The reflectance of the planar Si was greater than 35% due to the large difference between the refractive indices of the Si wafer and air. In contrast, the reflectance of the array of SiNWs was significantly lower, at no more than 7.2%, and was 3.0%, on average, over a wavelength range of 350−1000 nm. We may expect that the reflectance of the SiNW array will be less than that of the planar Si wafer because of light scattering within the array of SiNWs, which increases the effective optical path length, as scattered light may be incident on multiple surfaces^30^,^31^. Additionally, the array of SiNWs was sufficiently short (~200 nm) to minimize surface recombination while exhibiting superior anti-reflectance characteristics for the high efficiency solar cell application.

**Figure 5:**
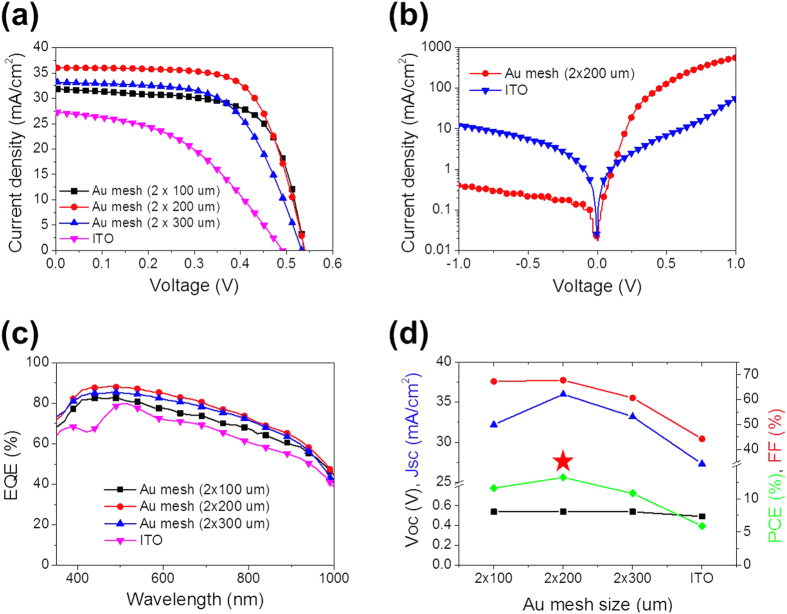
Photovoltaic properties of the hybrid solar cells. (**a**) The *J*–*V* characteristics of the hybrid solar cells with various front electrodes under illumination with AM 1.5 G light at 100 mW/cm^2^. (**b**) The *J*–*V* characteristics of the hybrid solar cells in the dark. (**c**) EQE spectra of the hybrid solar cells with various front electrodes. (**d**) A summary of the photovoltaic properties of the hybrid solar cells.

Figure 5a shows the current density—voltage (*J−V*) characteristics of the hybrid solar cells with Au mesh electrodes under illumination with simulated AM 1.5 G light with 100 mW/cm^2^ intensity. The *J-V* characteristics of an equivalent device with an ITO front electrode are also shown for comparison. The photovoltaic properties of *J*_*sc*_, *V*_*oc*_, *FF*, and PCE as a function of line pitch size are shown in Fig. 5d and summarized in Table 1. We can see that *V*_*oc*_ greater than 0.490 V was achieved, which indicates favorable properties of the heterojunction formed between SiNWs and PEDOT:PSS. The ITO cell exhibited the lowest PCE, 5.9%, which is attributed to the lower *V*_*oc*_ and *FF*. The PEDOT:PSS layer on the tip of SiNWs was very thin, so there was a greater probability of direct contact between the SiNWs and the ITO electrode due to the film-type morphology of the ITO. In other words, since the Au mesh has empty space (100–300 um) between the Au lines, there was less probability of direct contact between the SiNWs and the Au mesh electrode. Furthermore, the low pH of the PEDOT:PSS led to degradation in the performance of the ITO electrode following chemical stability tests, as shown in Fig. S2. This can be expected to lead to low shunt resistance (*R*_*sh*_) and high series resistance, thus reducing *V*_*oc*_ and *FF*. The steady-state *J−V* characteristics of the devices with the Au mesh and ITO electrode in the dark are shown in Fig. 5b. The *J−V* curves of the hybrid cells exhibit rectifying characteristics and significantly higher reverse saturation current (*J*_*0*_) by approximately one order of magnitude is achieved in the ITO cell. The decrease in *R*_*sh*_ and increase in *J*_*0*_ leads to a decrease in *V*_*oc*_ as follows:


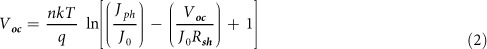


where *q*, *n*, *k*, *T*, and *J*_*ph*_ stand for electron charge, ideality factor, Boltzmann constant, temperature, and photogenerated current density, respectively. *FF* also can be described by the following expression:


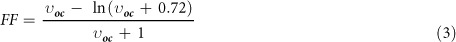



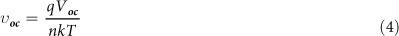


These relations clearly indicate that *FF* in the solar cells can be decreased by reducing *V*_*oc*_^32^. Furthermore, although the conductivity of the ITO electrode was sufficiently high for carrier collection, the ITO cell exhibited low short-circuit current density of *J*_*sc*_ = 27.3 mA/cm^2^ due to low optical absorption owing to the large reflectance and the filtering of the ITO electrode (see Fig. 2c). Compared with the ITO cell, the Au mesh cells exhibited a significantly improved *J*_*sc*_, which is attributed to the enhanced optical absorptance and carrier collection of the Au mesh cells (see the *J−V* curves shown in Fig. 5a). The best photovoltaic properties were obtained from the 200-μm-pitch cell, which had the most efficient carrier collection due to low sheet resistance of 16 Ω/sq and the highest optical absorptance (the peak transmittance of the 200-μm-pitch Au mesh was 90.9%). The PCE of this cell was 13.2%, along with *V*_*oc*_ = 0.539 V, *J*_*sc*_ = 36 mA/cm^2^, and *FF* = 67.8%. Because our samples had an identical device structure, except for the front electrode, all of the variation in the photovoltaic properties should be a direct result of the modifications of the front electrode.

To investigate the spectral response of the hybrid solar cells with different front electrodes, the external quantum efficiency (EQE) was analyzed using a xenon light source and a monochromator. The Au mesh electrodes led to a clear improvement in *J*_*sc*_ (see Fig. 5a), which corresponds to an increased EQE, as shown in Fig. 5c. The devices with Au mesh front electrodes exhibited enhanced spectral responses in the range 350–1000 nm compared with devices with ITO electrodes. The 200-μm-pitch cell showed the highest EQE, with an average value of 76.1% over a wide wavelength range of 350*–*1000 nm (although the optical absorption was weaker than that of the 300-μm-pitch cell, as shown in Figs 2c and 5c). Au mesh electrodes with a small line pitch diminish the probability of carrier recombination due to reduced charge-transport distance, resulting in increases in both *J*_*sc*_ and *FF*. As identified from electrical conductivity and optical transmittance analyses (see Fig. 2c), the mesh electrode with a small line pitch provided the highest electrical conductivity. For the 100-μm-pitch, however, *J*_*sc*_ was smaller due to the decreased optical transmission of the front electrode for active layer (see Fig. 5d). In contrast, a larger line pitch led to poorer charge carrier transport properties of the electrode. Thus, the poor performance of the hybrid solar cell with the 300-μm-pitch electrode is attributed to inefficient carrier collection. *FF* values also decreased with poorer carrier collection properties (i.e., an increased line pitch, as shown in Fig. 5d) because the carrier path was longer; thus, the series resistance was larger. Due to the increase in both *J*_*sc*_ and *FF*, the 200-μm-pitch cell provided a trade-off between these effects, leading to the highest PCE among the hybrid cells. This high PCE results from the improved optical absorption and efficient carrier collection at the Au mesh electrode, as identified from the EQE analysis (see Fig. 5c). These results clearly suggest that it is important to optimize the front electrode to fabricate highly efficient hybrid solar cells.

## Discussion

We have described the fabrication and characterization of SiNW/PEDOT:PSS hybrid solar cells with transparent and stable transfer-imprinted Au mesh front electrodes. The *J−V* and EQE results reveal that the Au mesh electrodes provide efficient charge carrier collection and high optical transmittance, which leads to a high PCE. A remarkable PCE of 13.2% was obtained by optimizing the line pitch of the Au mesh electrode. Although the Au meshes with a larger line pitch had higher transmittance, they did not collect charge carrier efficiently. In contrast, the Au meshes with a small line pitch led to worsening of optical transmission into the active layer. This work demonstrates that superior photovoltaic properties of hybrid solar cells can be realized by engineering the front electrode.

In addition, the EQE at short wavelengths (350*–*500 nm) indicates that the response to high-energy photons of all of our hybrid solar cells was improved relative to state-of-the-art Si/PEDOT:PSS hybrid solar cells^10^,^11^,^12^,^13^,^14^,^15^, demonstrating a low surface recombination in our SiNW/Al_2_O_3_/PEDOT:PSS architecture. In contrast, the EQE at long wavelengths gradually decreased with increasing wavelength. This is attributed to low-energy photons being absorbed farther down into the array of SiNWs, where minority carriers are not effectively diffused in the relatively thick (~525 μm thick) Si wafer. Therefore, we expect that a SiNW/PEDOT:PSS hybrid solar cell formed using a thin (<100 μm thick) Si wafer may offer further improvements in solar cell performance. The approach described here represents a platform for low-cost, highly efficient, flexible solar cells by adapting a thin Si substrate and roll-to-roll process.

## Methods

### SiNWs fabrication

The SiNW array was prepared on a Si(100) wafer (Czochralski-grown, *n*-type, 1–10 Ω·cm) using a simple, low-cost, metal-assisted chemical etching (MACE) method, in which metal deposition and chemical etching steps were performed independently to control the morphology of the SiNWs. The Si substrates were first cleaned using a standard wafer cleaning procedures, i.e., soaking in a 4:1 mixture of sulfuric acid (H_2_SO_4_) and hydrogen peroxide (H_2_O_2_) at 90 °C for 10 min to remove organic residues, followed by soaking in a 5:1:1 mixture of deionized (DI) water, H_2_O_2_, and hydrochloric acid (HCl) at 75 °C for 10 min to eliminate metal residues, and then soaking in 2% hydrofluoric acid (HF) for 1 min to remove oxides that were formed chemically during the cleaning process. The AgNPs were deposited onto the Si substrate using a galvanic displacement reaction with a mixed solution of AgNO_3_ (0.005 M) and HF (4.8 M). After immersion for 10 s in this solution, isolated AgNPs were formed. Chemical etching was then carried out using a mixture of DI water, HF (4.8 M), and H_2_O_2_ (0.6 M) at room temperature to form a vertically aligned array of SiNWs. The length of the SiNWs was controlled by varying the etching time. Following etching for 20 s, the SiNW samples were dipped in concentrated nitric acid (30 wt.%) for 20 min to remove the AgNPs.

### Transfer-imprinted metal mesh

A polyurethane acrylate (PUA) mold was replicated from an Si master using the replica molding method^33^. To increase the transfer ratio of the metal nanopattern from the mold, the surface of the PUA mold was coated with an nonstick layer (1*H*,1*H*,2*H*,2*H*-perfluorooctyl-trichlorosilane) prior to metal evaporation. A 150-nm-thick metal layer was then deposited on the surface of the PUA mold using an electron beam evaporator. Three different metals were investigated: Au, Ag, and Cu. The evaporation rate was 0.1 nm/s. To transfer the metal mesh layer from the PUA mold, a 1-μm-thick film of a transparent UV-curable adhesive (Norland Optical Adhesive 61, NOA 61) was spin coated onto a transparent substrate. To partially cure the UV-curable adhesive, the NOA 61-coated substrate was exposed to UV light for a few seconds using a 50 W mercury lamp. The PUA mold with the metal deposited layer was placed in contact with the partially cured substrate, and the assembly was completely UV cured with an applied pressure of 0.1 MPa for the sufficiently extended time. Finally, the PUA mold was peeled off, leaving the uniform metal mesh was on the transparent substrate.

### SiNW/PEDOT:PSS hybrid solar cells

Prior to device fabrication, the array of SiNWs was cleaned in a diluted HF (1%) solution for 1 min to remove any native oxide. The SiNWs were then immediately transferred to the ALD chamber (Lucida^TM^ D100, NCD) to form the Al_2_O_3_ passivation layer. A ~ 1.4-nm-thick Al_2_O_3_ film was deposited at 230 °C using trimethylaluminum (TMA) as a metal precursor and H_2_O as the oxygen source. Nitrogen was used as a carrier and purging gas. Filtered PEDOT:PSS (CLEVIOS PH1000) solution mixed with 5 wt.% dimethylsulfoxide (DMSO) and 0.2 wt.% Triton X-100 (Sigma-Aldrich) was spin coated onto both the SiNW array and the Au mesh electrode at 6000 rpm for 60 s, producing ~35-nm-thick layers. The PEDOT:PSS-coated Au mesh electrode and the array of SiNWs were directly pressed together and annealed using a hot plate at 150 °C for 10 min in a nitrogen atmosphere. To investigate the beneficial effects of the Au mesh electrode, a hybrid solar cell with an ITO electrode (Sigma-Aldrich, sheet resistance of 8.6 Ω/sq) was also prepared to provide a like-for-like comparison.

### Photovoltaic characterization

A 500 W xenon arc lamp with AM 1.5 G filters was used as the optical source to characterize the hybrid solar cells. The incident flux was confirmed using an NREL-calibrated solar cell (PV Measurements, Inc.). The *I*–*V* characteristics of the solar cells were investigated using a solar simulator (XES-502S, SAN-EL ELECTRIC) and a source meter (2400, Keithely) under illumination with 1-Sun light intensity (100 mW/cm^2^). The external quantum efficiency (EQE) was measured using a xenon light source with a spot size of 1 × 3 mm and a monochromator to select wavelengths in the range 350–1000 nm to investigate the spectral response of the solar cells.

## Additional Information

**How to cite this article**: Park, K.-T. *et al.* 13.2% efficiency Si nanowire/PEDOT:PSS hybrid solar cell using a transfer-imprinted Au mesh electrode. *Sci. Rep.* **5**, 12093; doi: 10.1038/srep12093 (2015).

## Supplementary Material

Supplementary Information

## Figures and Tables

**Table 1:** A summary of the photovoltaic characteristics of the hybrid solar cells with various front electrodes^a^.

**Front electrode**	***V*_*oc*_[V]**	***J*_*sc*_[mA/cm^2^]**	***FF*[%]**	***PCE*[%]**
ITO	**0.491**^b^ 0.487 ± 0.004	**27.3** 27.0 ± 0.3	**44.3** 43.3 ± 1.0	**6.0** 5.7 ± 0.3
Au mesh (300**-**μm pitch)	**0.538** 0.535 ± 0.003	**33.2** 32.8 ± 0.4	**60.7** 59.8 ± 0.9	**10.8** 10.5 ± 0.3
Au mesh (200**-**μm pitch)	**0.539** 0.535 ± 0.004	**36.0** 35.5 ± 0.5	**67.8** 67.0 ± 0.8	**13.2** 12.8 ± 0.4
Au mesh (100**-**μm pitch)	**0.538** 0.535 ± 0.003	**32.2** 31.8 ± 0.4	**67.3** 67.0 ± 0.3	**11.6** 11.4 ± 0.2

The parameters are the short-circuit current density (*J*_*sc*_), the open-circuit voltage (*V*_*oc*_), the fill factor (*FF*), and power conversion efficiency (PCE).

^a^Values are obtained by averaging four devices for each type.

^b^Numbers in bold are the maximum recorded values.
