# Supplementary material for: 13.2% efficiency Si nanowire/PEDOT:PSS hybrid solar cell using a transfer-imprinted Au mesh electrode
Source: Sci Rep. 2015 Jul 15;5:12093. doi: 10.1038/srep12093 (PMC4502511; doi:10.1038/srep12093)
Supplement: Supplementary Information [file srep12093-s1.pdf]

Supplementary Information

13.2% efficiency Si nanowire/PEDOT:PSS hybrid solar cell using a transfer-imprinted Au mesh electrode

Kwang-Tae Park[1]*, Han-Jung Kim[1]*, Min-Joon Park[2], Jun-Ho Jeong[1,3], Jihye Lee[1,3], Dae-Geun Choi [1,3], Jung-Ho Lee[2] & Jun-Hyuk Choi[1]

[1] Department of Nano Manufacturing Technology, Korea Institute of Machinery and Materials (KIMM), Daejeon, 305-343, Korea.

[2] Department of Chemical Engineering, Hanyang University, Ansan, 426-791, Korea.

[3]Department of Nano-Mechatronics, University of Science and Technology, Daejeon, 305-350, Korea

Correspondence and requests for materials should be addressed to J.H.C (junhyuk@kimm.re.kr)

Characterization of Au mesh electrodes

1. Chemical stability

Transparent electrodes should be able to withstand chemical attack in order to ensure durable device performance. For this reason, the chemical stabilities of Au mesh electrode and ITO electrode were evaluated by directly measuring sheet resistance after the samples were spin-coated with acidic PEDOT:PSS (CLEVIOS PH1000) solution. The PEDOT:PSS solution has a potential of hydrogen (pH) value of 1.5–2.5. And, it has been well-known that metal-based transparent electrodes can be etched when it is exposed to PEDOT:PSS solution. Figure S2 shows the changes in the sheet resistance of the Au meshes and ITO during chemical stability test. The change in resistance is expressed as R/R_0, where R_0 is the primary resistance and R is the measured resistance after chemical stability test. The R/R_0 value of the ITO electrode sample remarkably increased after 4 days. However, transfer-imprinted Au mesh electrode exhibited nearly constant R/R_0 value throughout the chemical stability test for 20 days. It is well-known that Au resists attacks by acid solution. Actually, the morphology of the Au mesh has not changed after this test (not shown in this article). These experimental results clearly indicate that Au mesh electrode could maintain the solar cell performances from the chemical attack.

2. Thermal stability

Highly robust transparent electrodes should be able to withstand high-temperature in the post-processing stages in order to ensure superior device performance. For this reason, the thermal stabilities of the Au mesh electrode and ITO electrode were evaluated by directly measuring sheet resistance when the all transparent electrode samples were placed on a high-temperature (200, 250, 300, and 350℃) hot-plate for 60 min. In this work, Cu and Ag mesh electrodes were

also prepared using the same method for comparison. Figure S3a–c shows the changes in the sheet resistance of the all transparent electrode samples during this test. The inset of the Figs. S3a–c shows the FESEM image of the Cu, Ag, and Au mesh electrodes on glass substrate after thermal stability test. Ag shown in Fig. S3a, the Cu mesh electrode showed stable electrical performance at 200℃ for 60 min. However, sheet resistance increased significantly when it was heated to 250℃. In order to investigate the cause of the performance degradation, FESEM and energy dispersive spectroscopy (EDS) analyses of Cu mesh electrode were performed before and after the thermal stability test. As shown in the inset of the Fig. S3a, the Cu mesh showed a practically negligible change in its morphology after annealing at 250℃. However, as shown in the Fig. S3d, EDS analysis confirmed the thermal oxidation of the Cu mesh electrode after thermal stability test. The Ag mesh electrode showed stable electrical performance at 200 and 250℃ for 60 min, as shown in the Fig. S3b. However, its sheet resistance increased rapidly when it was heated to 300℃. This is due to the morphology change of the Ag mesh after annealing at 300℃. Actually, as shown in inset of the Fig. S3b, annealing at 300℃ for 20 min caused the Ag mesh to break up. The ITO glass showed stable electrical performance at 300℃ for 60 min. However, as shown in Fig. S3c, the sheet resistance of the ITO glass slightly increased from 15.2 to 45.4 Ω/sq, even after exposure at 350℃ for 60 min. In contrast, the Au mesh electrode showed stable electrical performance at high temperature (350℃), reflecting its excellent thermal stability, as shown in the Fig. S3c. As shown in the inset of Fig. S3c, the morphology of the Au mesh has not changed after annealing at 350℃ for 60 min. This observation may be due to the higher thermal stability (higher melting point and low reactivity with air) of Au. This experimental result clearly indicates that thermal stability of the Au mesh electrode is higher than that of ITO electrode.

3. Structural and mechanical properties

Strong adhesion between metal-based transparent electrode and substrate is essential for practical use. For this reason, the fabricated various metal meshes on glass were subjected to a repeated adhesive tape test to evaluate the adhesion between metal mesh film and substrate. The adhesion tape test was done as follows: An adhesive tape (810, 3M) was firmly pressed onto the surface of the transparent electrodes and slowly peeled off. For comparison, a sample of ITO glass was also tested. Figure S4 shows the changes in sheet resistance of all transparent electrode samples during the test. As shown in Fig. S4, the sheet resistance of the ITO glass is almost the same as before the adhesion test. Similarly, the sheet resistance of the metal mesh electrodes remained unchanged after 10 cycles. This suggests that the adhesion between the metal mesh film and substrate was very strong even without any additional treatments.

The mechanical flexibility of transparent electrodes is very important to apply in flexible optoelectronic devices. For this reason, the Au mesh on polyethylene terephthalate (PET) film and ITO on PET film were rolled around a bending radius of 5 mm, and then unrolled at a speed of 1.0 cycle/s, after which the sheet resistance of each transparent electrode was compared to its initial value. Figure S5a shows the changes in sheet resistance of the Au mesh and ITO samples during bending-fatigue test. As shown in Fig. S5a, the ITO on PET film appeared to have the resistance increased sharply after only a few cycles. This is because ITO film on flexible substrate is brittle and easily cracks. Inset of Fig. S5a shows a microscope image of the ITO on PET film after this test. As shown in the inset of Fig. S5a, the ITO on PET film shows many cracks on its surface after mechanical flexibility test. In contrast, the Au mesh on PET film

showed stable electrical performance after 10,000 bending cycles. This indicates that the Au mesh on PET film possessed high mechanical flexibility. Figure S5b shows an image of a red light-emitting diode by using an electrically connected flexible and transparent Au mesh electrode.

4. Long-term stability

The long-term stability of transparent electrode is of prime importance for its practical use in optoelectronic devices. To evaluate the long-term stability of all transparent electrode samples to oxidation, metal mesh electrodes and ITO electrode were exposed to high humidity conditions (85~90% relative humidity, RH) at 80~85℃ for 30 days. In this work, Cu and Ag mesh electrodes were also prepared using the same method for comparison. Figure S6 shows the changes in sheet resistance of all samples during the long-term stability test. As shown in Fig. S6, the sheet resistance of Cu and Ag mesh samples increased significantly during this test. Especially, the change in sheet resistance of Cu mesh is more rapid than that of Ag mesh. The sheet resistance of the Cu mesh could no longer be measured after only 7 days. This observation may be due to the higher oxidation rate of Cu. It is well-known that Cu is more easily oxidized than Ag when they are exposed to same oxidative conditions. In contrast, the ITO electrode showed very stable electrical performance during long-term stability test. Au mesh electrode also showed stable electrical performance for 30 days, because Au does not readily react with the air and water. This suggests that the performance of Au mesh electrode could maintain for a longer time. These experimental results clearly confirm that the long-term stability of the Au mesh was comparable to that of ITO electrode.

In this study, we performed a comparative analysis of the durability of various metal (Cu, Ag, and Au) meshes, which were fabricated by using simple transfer imprinting method. As a result, we propose a highly robust and flexible Au mesh for alternative transparent electrode of ITO. The Au mesh electrode exhibited excellent optical and electrical properties. In addition, the Au mesh electrode exhibited the excellent long-term stability to oxidation as well as the chemical stability and strong adhesion, comparable to commercial ITO electrode. Surprisingly, the thermal stability and mechanical flexibility of the Au mesh electrode was higher than that of ITO electrode. From these experimental results, we strongly believe that our Au mesh electrode will be broadly applicable to high-performance optoelectronic devices.

Figure legends

Figure S1. FESEM image of the Au mesh patterns, with a line width of 2 µm and a line pitch of (a) 100 µm, (b) 200 µm, and (c) 300 µm. (d) A high-magnification SEM image of the Au mesh structure.

Figure S2. The sheet resistance of the ITO and Au mesh electrodes as a function of time during chemical stability tests.

Figure S3. The sheet resistance and morphology of (a) Cu, (b) Ag, and (c) Au mesh electrode samples during thermal stability tests. (d) The EDS results of the Cu mesh electrode before and after the thermal stability test.

Figure S4. Changes in the sheet resistance of the metal mesh electrodes and ITO electrode following adhesive tape tests.

Figure S5. (a) Changes in the sheet resistance of the ITO/PET and Au mesh/PET electrodes during mechanical flexibility tests. (b) A photograph of a red light-emitting diode operating with the flexible Au mesh electrode on a PET substrate.

Figure S6. Changes in the sheet resistance of the Cu, Ag, and Au meshes and the ITO electrodes during the long-term stability tests.

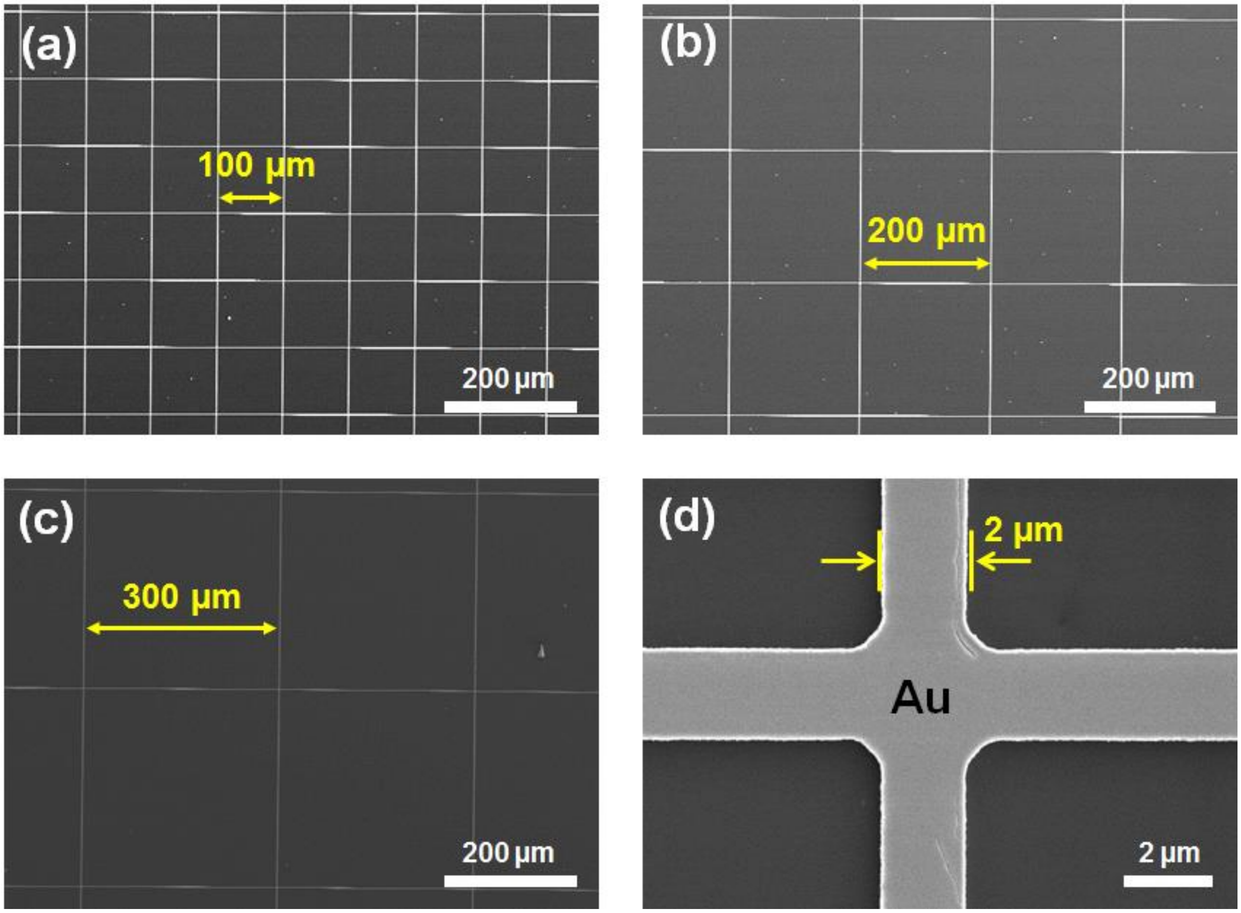

Figure S1

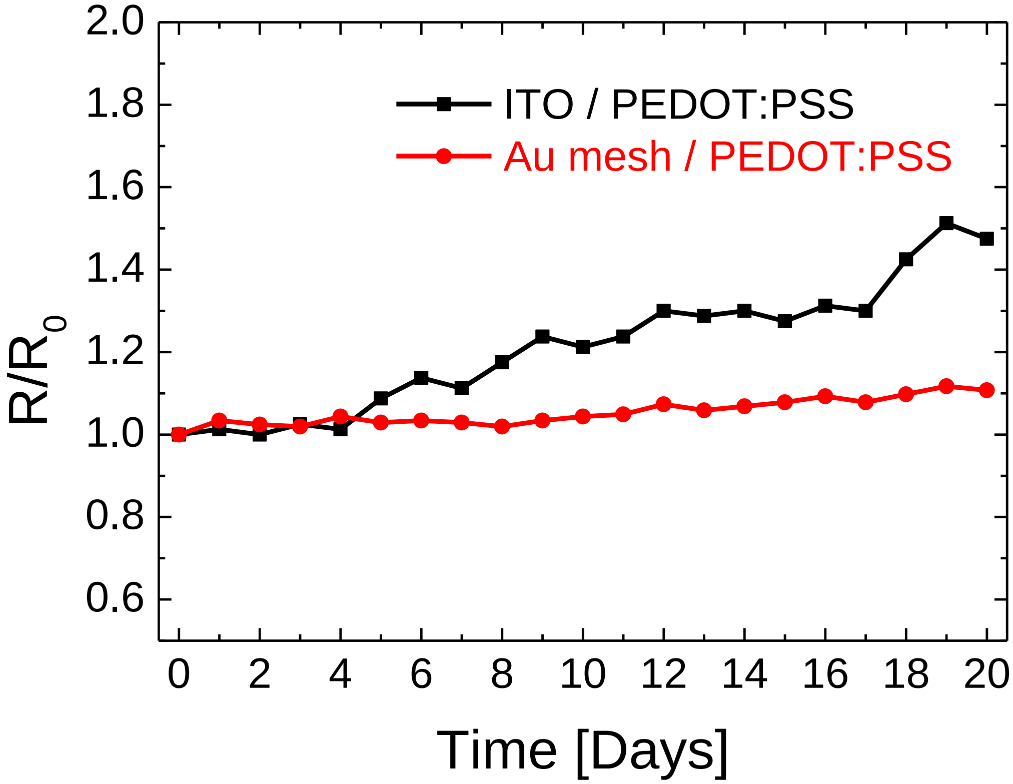

2.0
1.8
1.6
1.4
1.2
1.0
0.8
0.6
R/R0
ITO / PEDOT:PSS
Au mesh / PEDOT:PSS
0
2
4
6
8
10
12
14
16
18
20
Time [Days]

Figure S2

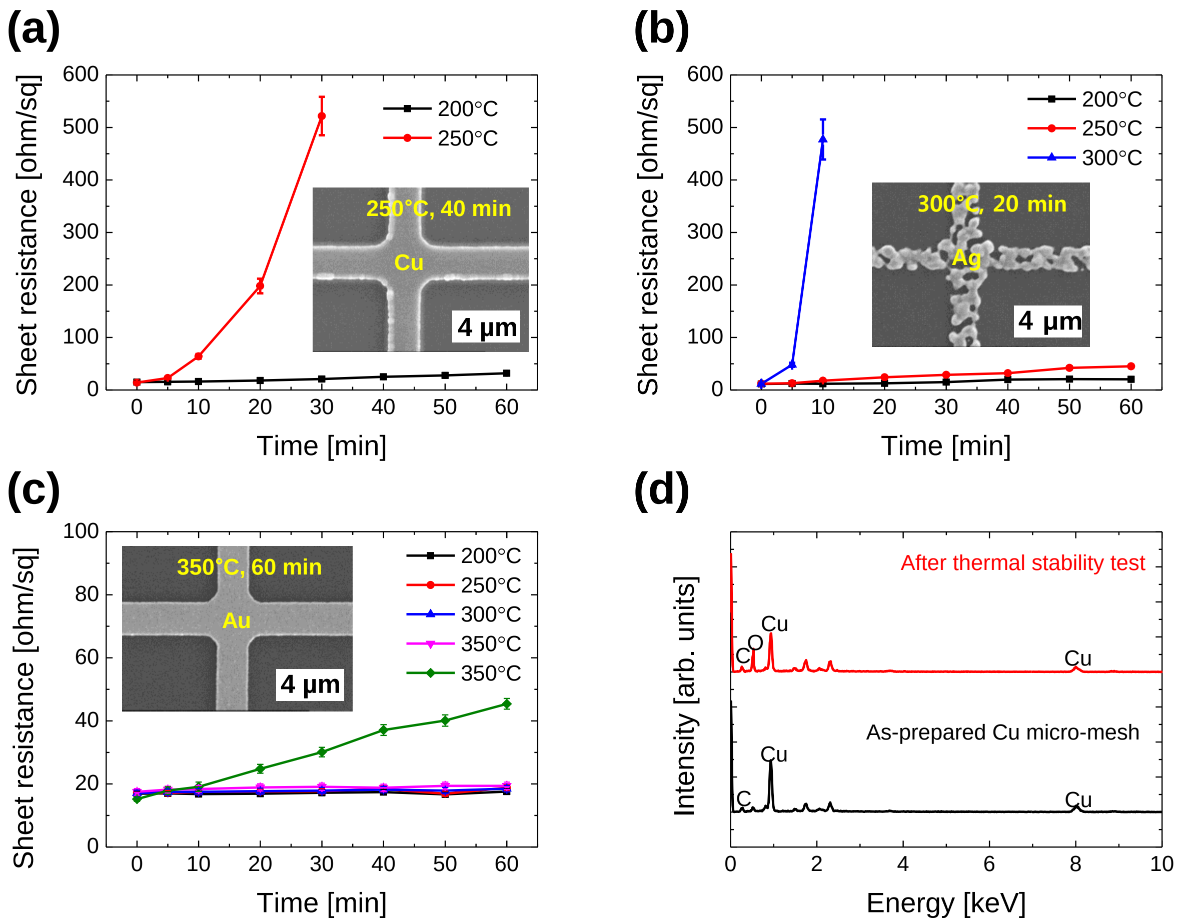

(a)
Sheet resistance [ohm/sq]
Time [min]
200°C
250°C
250°C, 40 min
Cu
4 μm
(b)
Sheet resistance [ohm/sq]
Time [min]
200°C
250°C
300°C
300°C, 20 min
Ag
4 μm
(c)
Sheet resistance [ohm/sq]
Time [min]
200°C
250°C
300°C
350°C
350°C
350°C, 60 min
Au
4 μm
(d)
Intensity [arb. units]
Energy [keV]
After thermal stability test
As-prepared Cu micro-mesh
C
O
Cu

Figure S3

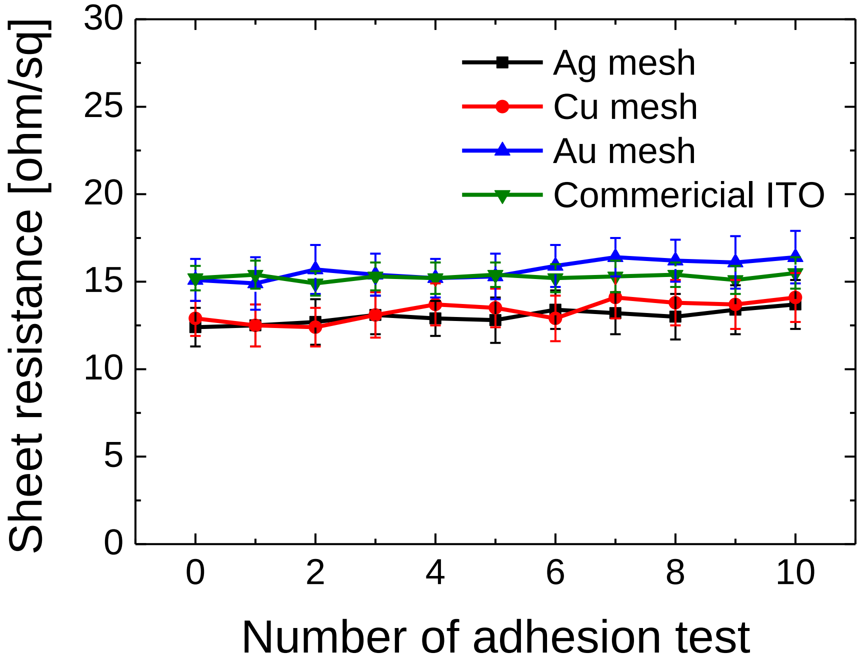
Sheet resistance [ohm/sq]
30
25
20
15
10
5
0
0
2
4
6
8
10
Number of adhesion test
Ag mesh
Cu mesh
Au mesh
Commericial ITO

Figure S4

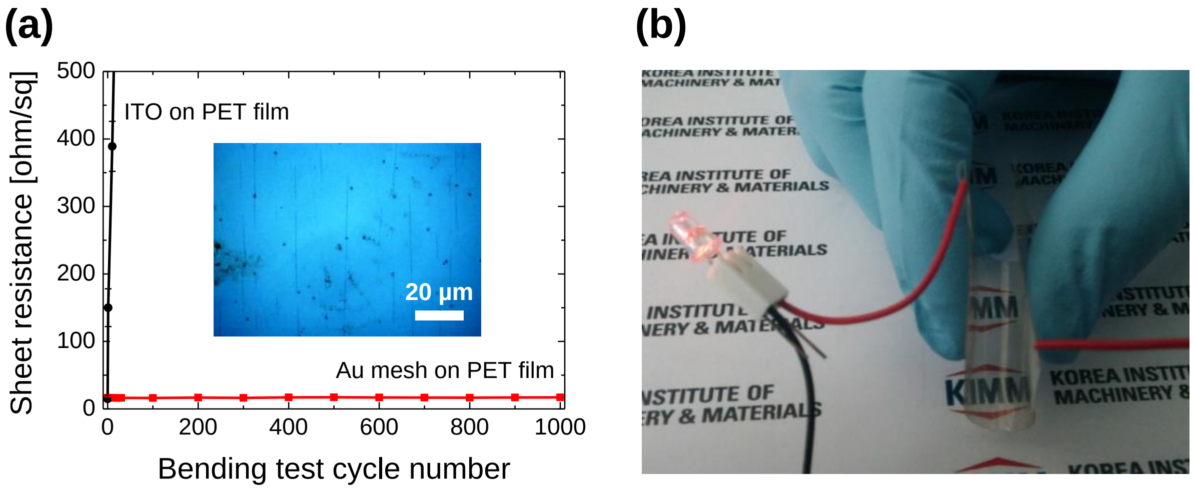
(a)
(b)
Sheet resistance [ohm/sq]
500
400
300
200
100
0
0
200
400
600
800
1000
Bending test cycle number
ITO on PET film
Au mesh on PET film
20 μm
KIMM
KOREA INSTITUTE OF MACHINERY & MATERIALS

Figure S5

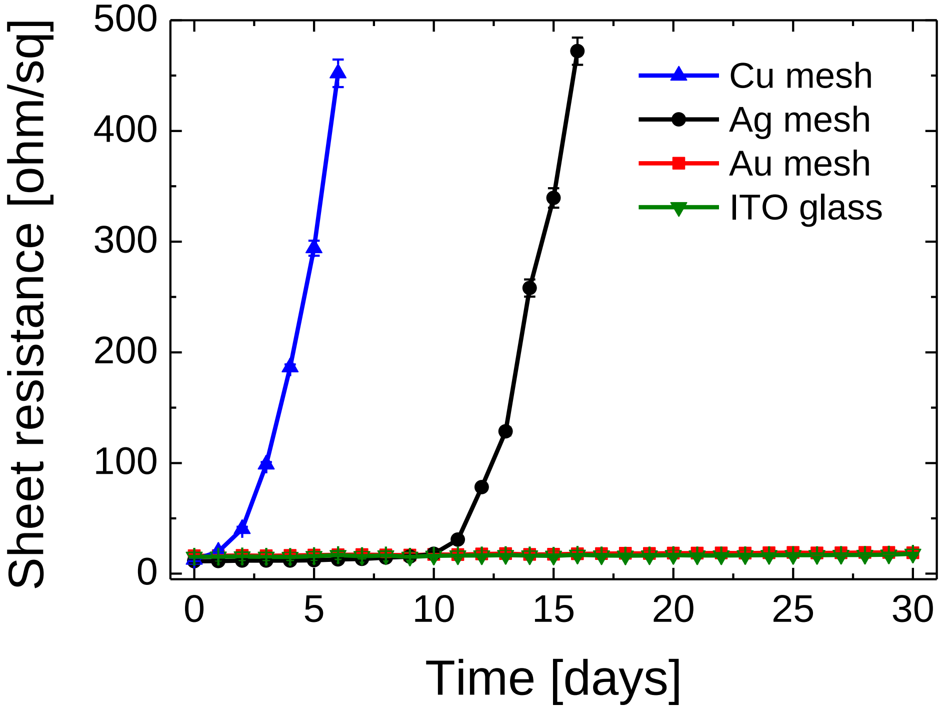

Sheet resistance [ohm/sq]
500
400
300
200
100
0
0
5
10
15
20
25
30
Time [days]
Cu mesh
Ag mesh
Au mesh
ITO glass

Figure S6
